# The WERO group stop smoking competition: main outcomes of a pre- and post- study

**DOI:** 10.1186/1471-2458-14-599

**Published:** 2014-06-13

**Authors:** Marewa Glover, Anette Kira, Dudley Gentles, Nathan Cowie, Chris Paton, Warren Moetara

**Affiliations:** 1 Centre for Tobacco Control Research, Social and Community Health, University of Auckland, Auckland, New Zealand; 2 George Centre for Healthcare Innovation, University of Oxford, Richard Doll Building, Old Road Campus, Roosevelt Drive, Oxford OX3 7LF, England; 3 Healthy Lifestyles Team, Northland District Health Board, Auckland, New Zealand

**Keywords:** Smoking cessation, Team quit and win contest, Indigenous people, Culturally informed

## Abstract

**Background:**

One potential promising strategy for increasing smoking cessation for Māori (Indigenous New Zealanders) and New Zealand resident Pacific Island people is Quit and Win competitions. The current uncontrolled pre and post study, WERO (WERO in Māori language means challenge), differs from previous studies in that it aims to investigate if a stop smoking contest, using both within team support, external support from a team coach and cessation experts, and technology, would be effective in prompting and sustaining quitting.

**Method:**

Fifteen teams, recruited from urban Māori, rural Māori and urban Pacific communities, competed to win a NZ$5000 (about €3,000, £2600) prize for a charity or community group of their choice. People were eligible if they were aged 18 years and over and identified as smokers. Smoking status was biochemically validated at the start and end of the 3 month competition. At 3-months post competition self-reported smoking status was collected.

**Results:**

Fourteen teams with 10 contestants and one team with eight contestants were recruited. At the end of the competition the biochemically verified quit rate was 36%. The 6 months self-reported quit rate was 26%. The Pacific and rural Māori teams had high end of competition and 6 months follow-up quit rates (46% and 44%, and 36% and 29%).

**Conclusion:**

WERO appeared to be successful in prompting quitting among high smoking prevalence groups. WERO combined several promising strategies for supporting cessation: peer support, cessation provider support, incentives, competition and interactive internet and mobile tools. Though designed for Māori and Pacific people, WERO could potentially be effective for other family- and community-centred cultures.

## Background

Several countries, including Australia, Finland, New Zealand, Singapore and Scotland, have reached low smoking prevalence (less than 20% of the general population) [1-5] and some have set goals to become smokefree. New Zealand’s near gold-standard tobacco control programme of regular tobacco tax increases, ongoing extensions to smokefree environments and tobacco product marketing bans, a free national Quitline and subsidised cessation products, has decreased daily smoking prevalence from 25% in 1996/1997 [2] to 15% in 2013 [6]. However, substantial socioeconomic and ethnic disparities in smoking persist: 15% of European New Zealanders are daily smokers, 38% of Māori (Indigenous New Zealanders, 15% of the population) and 23% Pacific Island people are daily smokers [2]. The New Zealand government’s aspirational goal is to be smokefree by 2025, defined as “a smoking prevalence of less than 5%, with tobacco being difficult to obtain and children not exposed to smoking” [7]. The 2018 midterm goal is to reduce the overall daily smoking prevalence to less than 10% and halve Māori and Pacific Island daily smoking prevalence to 19% and 11.5% respectively [8]. At the current cessation rate (supported and unsupported) of 3% per year [9], the Smokefree 2025 goals will not be reached. To achieve Smokefree 2025 and swiftly reduce smoking related harms, innovative strategies that can assist more smokers to more effectively quit will be needed.

The Ministry of Health’s key approach to reduce smoking prevalence is to increase quit rates by triggering more supported quit attempts more often [10]. To increase supported quitting among Māori, Pacific Island and low socioeconomic smokers a number of barriers to use of cessation support and products need to be addressed [11]. These barriers include a lack of knowledge, or incorrect knowledge of, cost or difficulty in accessing, low belief in the efficacy of, and low attractiveness of cessation products and services [12]. Designing public health interventions consistent with the cultural beliefs and practices of target populations is believed to lead to better receptivity, acceptance and salience of health information and programs [13].

Māori and Pacific Island cultures are family-centred with strong ties to their island and tribal identities which are maintained through culturally-based community-centres and groups and for Māori, language based schooling including pre-schools, and for Pacific, churches. Moreover, Māori and Pacific people have higher participation rates in organised competitions and events than New Zealand European people (44%, 41%, and 38% respectively) [14]. Māori cultural contests such as kapa haka (Māori performing arts) are well-attended and popular [15] and Pacific cultural competitions such as kilikiti (a form of cricket) claim high participation rates [16]. These contests incorporate inter-family, church or tribe competitiveness, are culturally-centred and encourage community participation-all factors identified as key components for effective recruitment and health intervention success for indigenous people [17].

Qualitative research with Māori and Pacific smokers identified that Quit and Win competitions are a potentially promising and attractive strategy for prompting cessation [18]. Although Quit and Win competitions vary, they often have these common elements: biochemical validation of smoking status, contest promoted in media and in participants’ community, winning a prize and support from health professionals [19]. However, the long-term efficacy of Quit and Win contests is inconclusive, with Quit and Win competitions delivering short-term quit rates ranging from 8% to 20% [19,20]. Furthermore, Quit and Win contests that rely on self-reported abstinence may suffer high deception rates and hence biochemical validation of abstinence claims are preferable [21]. The vast majority of Quit and Win competitions pitch individuals against each other [19]. A few workplace competitions published in the 1980s included teams, as opposed to individuals. All the studies had high end-of-competition rates for competition groups (39% [22], 22% [23], and 50% [24]). None of those studies achieved a significant higher quit rate at follow-up for the competition versus control group. However, in the area of obesity, research has found that group-based is more effective than individual-based competition and that the difference was maintained long term [25-27].

Despite the suggestion that social support influences intention to quit and maintenance of smokefree status [28] it has been underused in cessation interventions [29]. However, social support has been used and been found to have a positive influence on cessation when combined with Quit and Win competitions [30,31]. Contestants in Quit and Win contests who have a support person are more likely to quit and remain abstinent than those who do not have a support person [32]. One study found that 60% of their contestants utilised a designated support person, which reflects the importance of this type of support [32,33]. A pilot New Zealand contest that was run in 2000 also found that one of the factors associated with quit success was if the contestant had identified a support person [34]. The strategies for including social support have varied between different contests. For example, Pirie et al. [32] incorporated the social support into the contest, where the support person also got a prize if the smoker quit (but the support person was not a contestant themselves). A different strategy used by Croghan et al. [33] was to have two contests for one community – a Quit and Win contest for smokers and a Support Quit and Win for supporters.

### The current study

In order to reach the goal of Smokefree 2025, the New Zealand Ministry of Health’s key approach is to trigger more supported quit attempts. To date, as far as we can tell, Quit and Win competitions have not been adapted to include cultural elements, such as focusing on family or community groups and invoking existing inter-tribal rivalry to increase the attractiveness or relevancy for different ethnic groups [35]. By increasing attractiveness and thereby enrolment in cessation interventions it is possible to overcome some of the barriers to cessation, such as lack of knowledge and low attractiveness of cessation products. Furthermore, previous studies have identified the need for social support in Quit and Win contests, however, those studies incorporated a non-contestant to support a contestant. The previous studies that incorporated the support into a team contest are over 20 years old and were only tested in a work environment. The current uncontrolled pre and post study differs from those studies in that it aims to investigate if WERO (WERO in the Māori language means challenge), a stop smoking contest, using both within team support, external support from a team coach and cessation experts, and technology, would be effective in prompting quitting and increasing abstinence from smoking.

## Methods

### WERO - the intervention

WERO utilises several behaviour change strategies: incentives, competition, social support, behavioural therapy, pharmacological therapy and an interactive website and iPad application (app). Teams competed against each other for three months (31^st^ May- 30^th^ August 2012) to win a NZ$5000 (about €3,000, £2600, US$4000) prize to go to a charity or community group of their choice. Each team had to appoint their own coach (a non-smoker or ex-smoker) from their community. WERO co-ordinators assisted coaches to support their teams to stop smoking, to access cessation workers and pharmacotherapy and co-ordinators found in-kind support or spot prizes from within the community, such as free access to the public swimming pool, or retail vouchers from a sporting goods store. All WERO co-ordinators received training in how WERO works, their role, how to inform participants, obtain consent and collect data including exhaled carbon monoxide (CO) readings.

#### Technology

An interactive website and iPad app was used to strengthen the sense of team and inter-team competitiveness by making the smoking status of team members, and the team, publicly visible. Coaches updated participant smoking status weekly to the study website http://www.wero.me where each team had their own page.

The website also provided the participants with the opportunity to post to their team page and for supporters to post encouragement. Participants’ questions or comments, for example, about withdrawal symptoms and trigger situations, were answered and commented on by medical or cessation experts on the study team.

### Recruitment

WERO teams were recruited via community and health promotion workers from three organisations: Northland District Health Board (who recruited five rurally based Māori teams), Te Whānau o Waipareira, a Māori health and social services provider, in West Auckland, NZ’s biggest city (who recruited five urban Māori teams), and a Pacific Island churches healthy lifestyles programme, Enua Ola, in Auckland (who recruited five Pacific Island teams). Some teams included participants of mixed and other ethnicities. The cluster of teams are hereto forth referred to as Rural Māori, Urban Māori and Pacific. For each cluster, WERO co-ordinators were appointed to recruit teams from their community networks over six weeks (from mid-April until 31^st^ May of 2012). In Northland, this involved the co-ordinator approaching individuals in community groups such as sports clubs, kapa haka groups, marae (traditional Māori meeting locations) and small communities. For Pacific, the WERO co-ordinator approached key members of the church communities to recruit teams. Urban Māori teams were formed from staff within different business units within Te Whānau o Waipereira, a church group, a mums and babies group and a family group.

### Inclusion criteria

People were eligible to participate if they were aged 18 years and over, identified as smokers and had an exhaled CO rating greater than 6 parts per million (ppm), measured with a Bedfont Smokerlyser. The intervention was focused on Māori and Pacific Island people. However, it was a desired criterion, not an inclusion criteria per se.

### Exclusion criteria were

People who could not provide written consent, non-smokers or smokers whose exhaled CO was less than or equal to 6 ppm at entry were excluded from the study.

### Participants

Fifteen teams of 10 were to be recruited. Assuming 80% power, 5% significance, 1-sided test, no design effect, and a standard treatment 3 month quit rate of 24% (from the National Quitline), the study needed approximately 100 people to detect a target 3 month quit-rate of 35%. However, because the drop-out rate at follow-up is assumed to be high (50% from past studies), 150 smokers were recruited. For one team, two contestants stopped smoking prior to completing the eligibility screening and were hence deemed ineligible. Therefore, there were 14 teams with 10 contestants and one team with eight contestants, a total of 148 participants.

### Measures

At the end of competition and at follow-up, six months after the start of competition, contestants were asked *“In the last 7 days have you smoked a cigarette (even a puff)?”* However, interventions that use competitions or incentives suffer from high levels of deception [21]. Therefore, smoking status was biochemically validated at competition entry to determine eligibility, and at the end of the competition at 3 months to verify self-reported smoking status. Biochemical validation of smokefree status was undertaken using exhaled CO testing. For a contestant at the end of competition to be deemed to have quit smoking they had to have answered ‘No’ to the question *“In the last 7 days have you smoked a cigarette (even a puff)?”* and have an CO reading equal to or below 6 ppm (MacLaren et al. [36]). CO testing was chosen for pragmatic reasons, namely its low cost, ease of use and its availability for healthcare providers.

In addition, participants self-completed questionnaires, containing questions about gender, ethnicity, age, marital status and education level. Also to get feedback on the competition the following questions were included: In your opinion, what makes WERO successful? Tick box options were: Getting support to stop smoking; Getting to raise money for a good cause; Being in a competition; Stopping smoking in a team; and a free text option. Lastly, participants were asked to write about what did not work so well with the WERO competition and what could be done to improve it. The qualitative work adheres to the qualitative research reviews guidelines.

Lastly, qualitative interviews were conducted with co-ordinators and one coach from each cluster of teams at the completion of the 3 month data collection. The interviews included questions on intervention implementation, acceptability and satisfaction with the intervention, identification of barriers for effective implementation, and suggestions for improvement.

### Data analysis

The raw data was entered into an EXCEL spreadsheet and exported to SAS v 9.2 (Cary NY). Intention to treat analysis was used for all quit-rate calculations, that is, those lost to follow-up were assumed to be smoking. To determine the baseline predictors of those who quit at 6 months we used general estimating equation (GEE) analysis to account for correlation within teams and added a region variable (i.e., 5 teams each were nested within 3 clusters) into the model statement. Our model included ethnicity, gender, age (less than or equal to 26 years versus over 26 years), time to first cigarette (less than or equal to 30 minutes versus more than 30 minutes), and region. We chose the exchange structure (also known as compound symmetry) for the correlation matrix because it had a lower QIC score (Quasi-likelihood Information Criterion) than other structures.

The qualitative data was deductively analysed. Summary data were calculated for the participants’ quantitative interview questions.

### Ethical approval

Ethical approval was obtained through the Northern X Regional Ethics Committee, reference number NTX/11/EXP/308. Due to the public nature of the contest, participants smoking status was not kept confidential. However, all other data collected were confidential. All data were analysed anonymously. Informed consent was administered by the regional co-ordinators.

## Results

### Participant demographics

The age range of participants was 16 to 70 years old, with a mean of 38 years, 53% were Māori and nearly 60% were women (Table 1). On average contestants smoked 15.6 cigarettes per day. Many (71%) had their first cigarette within 30 minutes of waking up, indicating a high level of addiction. Thirty-seven per cent had never tried to quit before.

**Table 1:** **Participant demographics (% may not add to 100**% **because of rounding)**

**Variable**	**Count (%)**
**Ethnicity**	
Māori	78 (53%)
Samoan	32 (22%)
Cook Island Māori	4 (3%)
Tongan	10 (7%)
Niuean	7 (5%)
Other Pacific	4 (3%)
European	11 (7%)
Indian	2 (1%)
**Gender**	
Men	60 (41%)
Women	88 (59%)
**Age**	
15-24	18 (11%)
25-34	59 (36%)
35-44	33 (20%)
45-54	40 (24%)
55+	10 (6%)
Missing	6 (4%)
**Cigarettes smoked per week day**	15.6
**Time until first cigarette**	
Within 5 minutes	46 (31%)
6-30 minutes	59 (40%)
31- 60 minutes	16 (11%)
After 60 minutes	21 (14%)
Missing	6 (4%)
**Previous quit attempt**	
Yes	85 (57%)
No	55 (37%)
Missing	8 (5%)

### Main outcomes

At the end of competition the biochemically verified 24-hour quit rate was 36% (95% CI: 28%-44%) (Table 2). The 6 months self-reported quit rate was 26% (95% CI: 20%-34%). At competition end, loss to follow-up was 14% (21/148) and 19% (28/148) at 6 months.

**Table 2:** 7 day point prevalence at 3 and 6 months follow-up

	**3 months**		**6 months**	
	**Count**	**%**	**Count**	**%**
Not smoking	53	36% (quit-rate)^†^	39	26% (quit-rate)^‡^
Smoking	74	50%	81	55%
Did not complete question	1	0%	0	0%
Lost to follow-up (Dropout rate)	20	14%	28	19%
**Total**	**148**	**100%**	**148**	**100%**

^†^Biochemically verified.

^‡^Self-report only not biochemically verified.

Those aged 26 years and older were significantly more likely (OR = 1.99, 95% CI 1.08-3.64) to self-report being quit smoking at 6 months follow-up compared to younger participants (Table 3).

**Table 3:** GEE logistic regression model (n = 148) (accounting for correlation within teams and a region effect, exchange matrix and QIC = 175.096) with outcome those who quit cigarettes at 6 months follow-up and covariates: ethnicity, gender, age, and time to first cigarette

**Variable**	**Beta estimate**	**SE**	**P-value**
Māori versus Others	0.718	0.981	0.4640
Pacific versus Others	−2.043	1.630	0.2100
Female versus Male	0.323	0.234	0.1682
Age; Under or equal to 26 versus over 26 years	0.686	0.309	0.0264
Time to first cigarette (mins) 30+ versus under 30	0.084	0.171	0.6233
Pacific versus Urban Māori	3.648	2.040	0.0738
Rural Māori versus Urban Māori	0.725	0.441	0.1002

### Team and cluster of teams outcomes

The urban Māori teams had more participants that were 26 years old or younger, than the rural Māori or Pacific teams (Table 4). Pacific had a high rate (80%) of smokers who had never quit before, compared to 19% and 12% for the Māori urban and rural teams.

**Table 4:** Participants demographic by Region

	**Pacific count (col%)**	**Rural Māori count (col%)**	**Urban Māori count (col%)**	**Total count (col%)**
**Total**	50 (100%)	48 (100%)	50 (100%)	148 (100%)
**Ethnicity**				
Māori	0	43 (90%)	35 (69%)	78 (53%)
Samoan	30 (60%)	0	2 (4%)	32 (22%)
Cook Island Māori	1 (2%)	0	3 (6%)	4 (3%)
Tongan	10 (20%)	0	0	10 (7%)
Niuean	7 (14%)	0	0	7 (5%)
Other Pacific	2 (4%)	0	1 (2%)	3 (2%)
European	0	5 (10%)	6 (12%)	11 (7%)
Indian	0	0	3 (6%)	3 (2%)
**Gender**				
Men	30 (60%)	19 (40%)	11 (22%)	60 (41%)
Women	20 (40%)	29 (60%)	39 (78%)	88 (59%)
**Age**				
15-24	1 (2%)	4 (8%)	13 (26%)	18 (12%)
25-34	11 (22%)	16 (33%)	14 (28%)	41 (28%)
35-44	16 (32%)	8 (17%)	9 (18%)	33 (22%)
45-54	20 (40%)	12 (25%)	8 (16%)	40 (27%)
55+	2 (4%)	6 (13%)	2 (4%)	10 (7%)
Missing	0	2 (4%)	4 (8%)	6 (4%)
**Cigarettes smoked per day**				
Week day	16 (IQR* = 12)	17 (IQR* = 12)	13 (IQR* = 12)	16 (IQR* = 12)
Weekend day	16 (IQR* = 12)	20 (IQR* = 15)	15 (IQR* = 10)	17 (IQR* = 10)
**Time to first cigarette**				
1 = within 5mins	14 (28%)	14 (29%)	18 (36%)	46 (31%)
2 = 6–30 mins	25 (50%)	17 (35%)	17 (34%)	59 (40%)
3 = 31–60 mins	5 (10%)	5 (10%)	6 (12%)	16 (11%)
4 = after 60mins	6 (12%)	8 (17%)	7 (14%)	21 (14%)
Missing	0	4 (8%)	2 (4%)	6 (4%)
**Previous quit attempt**				
Yes	9 (18%)	35 (73%)	41 (82%)	85 (57%)
No	40 (80%)	9 (19%)	6 (12%)	55 (37%)
Missing	1 (2%)	4 (8%)	3 (6%)	8 (5%)

*IQR = inter-quartile range (i.e., The75^th^ minusthe 25^th^percentiles).

The number of quitters per team ranged from zero (no quitters) to ten (all quit). Three teams had no quitters and one team had ten quitters. The average number of quitters per team was 3.5 and the median number of quitters per team was 3. The Pacific teams had the highest overall quit rate at both end of competition and at 6-months follow-up (Table 5). However, the winning team, with 10 out of 10 quitters, were a rural Māori team. The urban Māori teams had substantially lower quit rates at both end of competition and 6-months follow-up than the rural Māori or Pacific teams.

**Table 5:** Quit and Dropout rates by cluster of teams

	**Pacific**		**Rural Māori**		**Urban Māori**	
	**3-month**	**6-month**	**3-month**	**6-month**	**3-month**	**6-month**
Quit rate	46% (23/50)	36% (18/50)	44% (21/48)	29% (14/48)	18% (9/50)	14% (7/50)
Dropout rate	4%	4%	13%	11%	26%	42%

### Co-ordinators’, coaches’ and contestants’ views

All coaches and co-ordinators expressed great satisfaction and acceptability of the intervention, including a coach from and the co-ordinator of the Māori urban teams that did not have as many quitters.

I think it’s [WERO] great!… It’s going to help get people going and it’s going to motivate people to have a go. (Rural Māori coach)

The idea [WERO] is absolutely fantastic… for us Pacific and Māori people because … one person will look at the other person, you know and copy what he does, one quits and the other one will follow. (Pacific co-ordinator)

It was identified that the concept of WERO could be applicable in other areas also.

I think it was a good platform for us to look at other things as well, like a good model I should say for us. You can use it in any environment, or any group organisation (Rural Māori Co-ordinator)

A number of contestants provided very positive feedback.

Thanks to this programme cause it helped me stop smoking and working as a team was great.

I have no idea how you could improve the comp. But I do know, that, this kaupapa [programme] works. I have been smoking longer than most of the members in my haka roopu [kapa haka group] and now I'm smokefree.

The most common activities that the contestants found helpful were support, from each other, the team coach and WERO co-ordinators, and getting together and competing against other teams (Table 6a). The rural Māori teams particularly found the within team support and support from coaches and co-ordinators helpful, while more of the Pacific teams selected getting together as helpful. Only 7% of participants didn’t take part in the activities, nearly all from the urban Māori teams, and only 2% did not find any of the activities helpful, all from the urban Māori teams.

**Table 6:** Acceptability and success of WERO

	**Overall**		**By group**					
			**Pacific**		**Urban Māori**		**Rural Māori**	
	**Count**	**%**	**Count**	**%**	**Count**	**%**	**Count**	**%**
**a) Helpful activities**								
Supporting each other	50	39%	14	29%	13	35%	23	53%
Support from our team coach	48	38%	14	29%	12	32%	22	51%
Support from WERO co-ordinator	48	38%	18	38%	5	14%	25	58%
Support from a stop smoking advisor	10	8%	1	2%	2	5%	7	16%
Support from whānau	6	5%	0	0%	0	0%	6	14%
Socialising together	38	30%	20	42%	7	19%	11	26%
Meeting together	42	33%	21	44%	5	14%	16	37%
Sharing our stories	26	20%	8	17%	6	16%	12	28%
Making a commitment to our group	35	27%	9	19%	7	19%	19	44%
Competition within team	16	13%	6	13%	4	11%	6	14%
Competing against other teams	40	31%	20	42%	9	24%	11	26%
Don't know	6	5%	2	4%	2	5%	2	5%
I did not take part in any activities	9	7%	0	0%	7	19%	2	5%
I did not find any of these helpful	2	2%	0	0%	2	5%	0	0%
**b) Successful WERO elements**								
Selected at least one	116	91%	45	94%	30	81%	41	95%
Didn’t select any option	34	27%	3	6%	7	19%	2	5%
Said “nothing was successful”	3	2%	0	0%	3	8%	0	0%
**Options selected**								
Getting support to stop smoking	74	58%	16	33%	21	57%	37	86%
Raising money for a good cause	55	43%	21	44%	8	22%	26	60%
Being in a competition	45	35%	13	27%	11	30%	21	49%
Stopping smoking in a team	69	54%	22	46%	14	38%	33	77%

Over 90% of participants selected one of more options as successful, 27% of participants did not select any options as successful and three participants stated that nothing was successful with WERO (Table 6b). The most commonly selected options were getting support to stop smoking and stopping smoking in a team.

Coaches, co-ordinators and contestants (Table 6a) identified that between-teams contest was most important.

Oh that’s [between team competition] really important… if all the regional teams know each other then that would make it a bit more competitive. (Urban Māori co-ordinator)

Yeah, I think it was very important to know that there are other teams as well in the competition and for everyone to try and stop smoking (Pacific coach)

Competing against teams from other clusters and Māori versus Pacific was also important:

We always saw it as a Tai Tokerau [Northland] vs Tāmaki [Auckland] thing. (Rural Māori Co-ordinator)

Only 31 contestants provided any criticism of WERO. The most common critiques of WERO, from both coaches, co-ordinators and contestants was difficulty in getting team members together. Some contestants reported that there was lack of commitment (n = 4) or support (n = 4) from team members, and difficulty in attending meetings (n = 3). Coaches and co-ordinators also stated difficulties getting some teams together.

Towards the end I just got really hoha (annoyed) cause it was a real hassle trying to get them there but they were really over it. (Urban Māori coach)

Challenges. It is getting everybody together to meet. (Rural Māori coach)

Even though they were keen to participate and be involved, it was really hard to get them all together in one place…. One of the things that worked to do that was when they had their wānanga [educational training workshops] every month. (Rural Māori co-ordinator)

Another criticism from coaches was the limited time prior to the start of the contest for coaches to prepare.

It happened all a bit quickly; in the beginning we really didn’t have time to sit down and organise sessions etc. properly. (Rural Māori coach)

Probably the timing as well. More warnings and that would be really good. (Pacific coach)

An Urban Māori coach thought that the contest wasn’t long enough, while the rest of the coaches and co-ordinators thought that the length was just right.

It’s probably not long enough cause even if you’re just working with people and you know you’ll have a drop off period maybe I think 3 months is too short. (Urban Māori coach)

I think 3 months is perfect… if it’s earlier, it wouldn’t of given heaps of people a chance to become a non-smoker. So I think 3 months is good. (Urban Māori co-ordinator)

There were also some technical difficulties. Three contestants had no access to technology (n = 3). Some teams didn’t have internet access.

So actually another challenge was a lot of people didn’t have internet. (Urban Māori coach)

There were also some concerns about the CO testing:

The carbon testing thing, I don’t think it was accurate (Urban Māori coach)

Only 31 participants provided any suggested improvements. The most common suggestion (n = 11) was for more prizes, for example, some prize for all who managed to get smokefree, smaller prizes at regular intervals of the competition and gifts for all participants.

Maybe even if all of the team would get something. That that would be… even if just a small prizes for every team and they would be very appreciative. (Pacific Co-ordinator)

Contestants, co-ordinators and coaches suggested more activities, within teams, with WERO support staff and with other teams.

We could have all met one another, scoped out our competition but we’re all disconnected. (Urban Māori coach)

## Discussion

WERO achieved a high quit rate among a high smoking prevalence group. Compared to the national quitting rate (19% for Māori and 14% for Pacific for supported quitting at 6-months follow-up [37]), and previous Quit and Win contests (for example [19]) the WERO end of competition quit rate was high. It should be noted that the design of WERO limits the comparability with previous Quit and Win contests [21-24], since those studies were conducted with more rigorous design. However, given the high smoking prevalence and non-significant reduction in smoking prevalence for Māori (remaining at 41%) or Pacific Island people (remaining at 26%) [2], WERO appeared to also be successful in achieving a high 6-month self-reported follow-up quit rate. Contestants, team coaches and WERO co-ordinators found the intervention acceptable. They particularly liked the group aspect and the inter-team competition. Recruitment of Indigenous people into research interventions can often be problematic [38], but was successful over a short recruitment period for WERO. WERO was informed by Māori culture, but findings should be of interest to other cultures who are family-centred and competitive also. This is of particular relevance, since many other countries, such as the USA, Canada and Australia, have disproportionately high smoking prevalence among their Indigenous, Pacific and Asian people. Furthermore, previous research has identified that friendly competition can be an effective element in health interventions for Indigenous people [39].

The findings from this study support previous work [30-33,40-42] that highlighted the importance of social support in Quit and Win contests. However, the difference between WERO and, for example, Croghan, O’Hara, Schroeder, et al. [33] was that the team members were both smokers *and* supporters. Previous workplace Quit and Win team contests [22-24], achieved high end-of-competition quit rates, but were not successful in achieving significantly higher follow-up quit rates. One potential explanation for the high 6-month follow-up quit rate in WERO, is that the teams were mostly made up of existing groups with close ties, who may have been able to continue to support each other. The use of technology in WERO potentially increased the support contestants received from their team members and also cessation experts. It also provided a forum where contestants could engage with other teams, the competitive element could be built upon, and contestants could gain a sense of being part of the efforts of many others to quit. Māori are early adopters of technology: 78% of Māori have access to a computer with internet and 87% have a cell phone or PDA [43]. The number of online health interventions is rapidly increasing and is showing promise in changing behaviour [44].

WERO has subsequently been funded to run a series of country wide competitions that will be made up of smaller regional or inter-regional competitions. There is no limit to the total number of teams that can register in concurrent competitions, thus enabling mass quitting to occur. Further, WERO has been designed to be used by organisations at a number of levels from health providers down to local providers and community groups. WERO is a voluntary removal of demand from tobacco products.

### Strengths and limitations

The main strength of this study was that the intervention was informed by the needs of the target audience, identified in previous research by the authors. The intervention was innovative in its use of new technology (e.g. apps) applied at a community level. This fitted with the New Zealand Tobacco Control Research Tūranga brief for research to be innovative, pragmatic, able to demonstrate a strong link to rapid prevalence reduction, be relatively short in duration from conception to delivering of results and have the potential to be scaled up. The requirement to conduct shorter studies imposes some limitations. Strongly powered randomised controlled trials, for instance, are beyond the scope and budget available to the Turanga. Hence the short-term pre and post study non-randomised design used here. One limitation of the research design, is that it was not possible to control for confounders, such as the impact of individual co-ordinators or coaches. The design also limits the comparability of WERO to previous Quit and Win contests [19] and previous workplace studies [19,22-24], since those studies were conducted in different countries and with more rigorous design. Our study also lacked a control group so it is difficult to ascertain how much of the high quit rate was due to the external environment rather than the intervention itself. However, as far as we are aware, there were no major significant tobacco control policies, such as tax increases, during the intervention period. End-of-competition smoking status in this study was ascertained using CO tests. Given that CO levels only assess smoking in the preceding nine hours, it is possible that the quit rates reported in this study are an overestimate. Cotinine tests would have provided a more reliable assessment of smoking cessation, however, it was not feasible in this study due to costs. Lastly, 6-month follow-up relied on self-report and was not biochemically verified. This could also potentially overestimate the quit rate.

### Future work

The limitations of this study highlight the need for future controlled trials with longer follow-up times of team based cessation contests like WERO. The survey and interview data suggests that the group element and social support were particularly satisfactory for the contestants, while the prize structure may not have been optimal. While some teams thought that it was ideal to donate the prize to a charity, others would have liked to receive the prize themselves. The WERO intervention comprised several behaviour change strategies but the design of the study precluded independent assessment of the relative contribution of each. Future controlled studies need to be conducted that allow for assessment of the individual elements, as well as the influence of co-ordinators or coaches. It would be useful to investigate if WERO would be as effective with other populations, for example Indigenous populations in other countries, or pregnant smokers. It is also possible that this intervention could be modified to trigger change in other health related behaviours, such as physical activity and nutrition. Lastly, it would be useful to conduct future work to establish cost-effectiveness and the cost per quitter of running this type of intervention.

## Conclusion

If NZ is to reach its smokefree nation goal by 2025, innovative tobacco control strategies that can induce rapid reduction in smoking prevalence within existing health budgets are needed. Groups based quit competitions, like WERO, offer one such solution.

## Competing interests

The authors declare that they have no competing interests.

## Authors’ contributions

MG conceived of the study and was involved in all aspects of the study. AK participated in design of study, review of literature and critical revisions of the manuscript. DG and AK conducted the analysis. NC, CP and WM participated in design of study, website and iPad application and development of other study materials and documents. They provided input into and critical review of the manuscript. All authors read and approved the final manuscript.

## Pre-publication history

The pre-publication history for this paper can be accessed here:

http://www.biomedcentral.com/1471-2458/14/599/prepub
